# Competitive Exclusion of Intra-Genus *Salmonella* in Neonatal Broilers

**DOI:** 10.3390/microorganisms9020446

**Published:** 2021-02-21

**Authors:** Megan Pineda, Michael Kogut, Kenneth Genovese, Yuhua Z. Farnell, Dan Zhao, Xi Wang, Allison Milby, Morgan Farnell

**Affiliations:** 1 Department of Poultry Science, Texas A&M AgriLife Research, College Station, TX 77843, USA; meganpineda@tamu.edu (M.P.); yfarnell@tamu.edu (Y.Z.F.); dz137@tamu.edu (D.Z.); phdwangxi@outlook.com (X.W.); a.milby@tamu.edu (A.M.); 2 Southern Plains Agricultural Research Center, Agricultural Research Service, United States Department of Agriculture, College Station, TX 77843, USA; mike.kogut@ars.usda.gov (M.K.); kenneth.genovese@ars.usda.gov (K.G.)

**Keywords:** broiler, *Salmonella* Kentucky, *Salmonella* Typhimurium, *Salmonella* Enteritidis, colonization resistance, poultry, immune response, cytokine

## Abstract

Salmonellosis is a zoonotic infection caused by *Salmonella enterica* serotypes contracted from contaminated products. We hypothesized that competitive exclusion between *Salmonella* serotypes in neonatal broilers would reduce colonization and affect the host immune response. Day of hatch broilers were randomly allocated to one of six treatment groups: (1) control, which received saline, (2) *Salmonella* Kentucky (SK) only on day 1 (D1), (3) *Salmonella* Typhimurium (ST) or *Salmonella* Enteritidis (SE) only on D1, (4) SK on D1 then ST or SE on day 2 (D2), (5) ST or SE on D1 then SK on D2, and (6) SK and ST or SE concurrently on D1. *Salmonella* gut colonization and incidence were measured from cecal contents. Livers and spleens were combined and macerated to determine systemic translocation. Relative mRNA levels of interleukin-1β (IL-1β), IL-6, IL-10, IL-18, and gamma interferon (IFN-γ) were measured in cecal tonsils and liver to investigate local and systemic immune responses. When a serotype was administered first, it was able to significantly reduce colonization of the following serotype. Significant changes were found in mRNA expression of cytokines. These results suggest competitive exclusion by *Salmonella enterica* serotypes affect local and systemic immune responses.

## 1. Introduction

*Salmonella enterica* is a foodborne pathogen that causes an estimated 1.2 million human infections annually in the United States [1]. There are more than 2600 *Salmonella enterica* serotypes which can infect a wide range of vertebrate species, but fewer than 100 serotypes cause the majority of human infections [2,3]. Infected poultry may be asymptomatic of clinical disease but continue to shed zoonotic subspecies into the environment causing gastroenteritis and systemic infections in humans [4,5]. *Salmonella enterica* Typhimurium (ST), Kentucky (SK), and Enteritidis (SE) are in the top 5 common isolates found in contaminated chicken [4,6,7]. Reducing *Salmonella* colonization of poultry would benefit agricultural and public health sectors by decreasing medical costs and lowering cases of human infections.

In 2014, the US Department of Agriculture (USDA) Food Safety and Inspection Service determined that SK, SE, and ST were isolated from 60.8%, 13.6%, and 7.7% of young chicken carcasses, respectively [3]. *Salmonella enterica* serotype Kentucky (SK) is the most prevalent serotype isolated from contaminated poultry carcasses; however, only 0.14% of human clinical disease was reported with this bacterium [8]. In 2016, SE and ST were confirmed from 16.8% and 9.8% human Salmonellosis cases, respectively [8]. These *Salmonella* are prevalent in poultry; however, only SE and ST are considered a major food safety concern when compared to SK [3].

Neonatal chicks are more susceptible to foreign bacteria, such as *Salmonella*, due to their lack of mature intestinal microbiota [9]. Initial invasion in the gastrointestinal tract leads to increased expression of chemokines, cytokines and an influx of heterophils and macrophages [10,11,12]. *Salmonella* Typhimurium infection significantly decreased jejunum villus height from the host’s inflammatory response due to the influx of heterophils in one-day-old chicks [13,14]. *Salmonella* can hide, multiply and survive in macrophages leading to persistence [15]. Disease tolerance occurs as *Salmonella* is then able to persist in the gut of chickens without severe clinical signs [16].

Intestinal direct colonization resistance is the inability of foreign ingested bacteria to colonize due to host bacteria [17]. Mechanisms can include nutrient competition or active antagonism, such as bacteriocins [18,19]. Rantala and Nurmi used mature chicken intestinal bacteria to reduce colonization of *Salmonella* Infantis in chicks [20]. Oral administration of attenuated ST given to one-day-old chicks has been shown to competitively exclude future colonization of intestinal ST when it is again administered 24 h later [21]. Methner and colleagues challenged chicks with various *Salmonella* serotypes and found greater inhibition occurred between isogenic strains [22]. Yang and colleagues determined intra-genus competitive exclusion occurs between ST and SE when administered 24 h apart in neonatal broilers [23]. All are examples of ways colonization resistance can be used to exploit competitive exclusion in order to reduce *Salmonella* colonization in chicks.

Indirect colonization resistance occurs through microbiota-stimulated host immunity and immune cell interactions. These include an enhanced mucosal barrier by production of mucus, short-chain fatty acids, such as butyrate or acetate, and host antimicrobial peptides from resident Paneth cells [24]. Characterization of a broiler’s immune response during a *Salmonella* infection can be measured by cytokine gene expression. An early localized inflammatory response includes an influx of heterophils and macrophages [25]. Interleukin-1β (IL-1β) is a pro-inflammatory mediator and expression is increased in response to bacterial, viral, and parasitic infections [26]. The expression of other pro-inflammatory cytokines, such as IL-6 enhance protection during ST infection by inducing acute phase protein synthesis and are important in further stimulating a T_H_1 host immune response [27,28]. Interleukin-18 is a member of the IL-1 family and is produced by macrophages in response to lipopolysaccharide [29,30]. When IL-18 is in the presence of IL-12, gamma interferon (IFN-γ) production is upregulated in T_H_1 and NK cells [30]. Chicken IFN-γ is a macrophage-activating factor and is crucial in response to intracellular pathogens by inducing a cell-mediated T_H_1 response [31,32,33]. The function of IL-10 is to induce immunoregulatory effects, such as downregulating the production of pro-inflammatory cytokines [34]. The cecal tonsils are composed of lymphoid follicles that contain multiple immune cells [35]. The liver is important for immune function, containing Kupffer and other immune cells, synthesizing cytokines, chemokines, and acute-phase proteins in response to infection, trauma or stress [36]. Acute phase proteins, such as mannan binding lectins, can activate phagocytes and modulate cytokine expression [37]. Therefore, cytokine mRNA expression can be measured in chicken cecal tonsils and livers to measure the host immune interaction with *Salmonella* as a local or systemic infection [38]. The expression of cytokines can correlate with the presence of pathogens in the gut; however, mRNA expression may not always indicate actual protein synthesis [38].

*Salmonella* Kentucky (SK) is the number one isolated serotype in poultry but causes a low incidence (0.14% cases) of clinical disease in humans [8]. Fricke and colleagues screened *Salmonella enterica* strains for avian pathogenic plasmid uptake from *Escherichia coli* to screen for virulence evolution and host adaptations [39]. Evidence of these plasmids was predominantly found in SK but not in other strains of *Salmonella*, which could give an advantage to SK to cope with stress factors and competition [39]. Cheng and colleagues showed SK persisted until the end of their challenge on day 36 compared to ST colonization which had fallen below the level of detection by day 15 [40]. Prevalence of SK in the ceca, over other serotypes, could be further explained at the molecular level, such as increased transcription of regulatory protein, RNA polymerase (Rpos) [40]. A sigma factor, such as Rpos, initiates transcription in stress response genes, so if it were elevated it would allow for greater proliferation of bacteria [41]. Since SK is the predominantly isolated serotype, we believe this serotype may be used to exclude and reduce other *Salmonella* as a live vaccine candidate.

We hypothesized that an initial infection by SK can reduce intra-genus serotypes SE and ST by competitive exclusion. Incidence of SK in the poultry industry is increasing [39]. Understanding how these serotypes interact is necessary, but there is little information available. Furthermore, we attempted to characterize the host immune response by measuring cytokine gene expression in cecal tonsils and livers during a co-infection.

## 2. Materials and Methods

### 2.1. Experimental Birds

Day-of-hatch, male broiler chicks obtained from a commercial hatchery were placed on clean pine shavings in floor pens with an environmentally controlled and age-appropriate climate in animal biosecurity level 2 rooms [42]. Chicks were provided ad libitum access to water and a balanced unmedicated starter ration that met or exceeded industry recommendations for nutrition [43]. Upon arrival, chick tray papers were cultured to confirm that the chicks were *Salmonella*-negative. Each cohort of birds were placed in pens sized 3.7 m × 2.7 m. 

### 2.2. Bacterial Strains and Growth Conditions

All serotyped isolates (Table 1) were obtained from USDA-ARS (College Station, TX, USA) and were stored at −80 °C. *Salmonella* Kentucky (SK), *Salmonella* Enteritidis (SE), and *Salmonella* Typhimurium (ST) were passaged 3 times every 8 h in sterile tryptic soy broth (TSB; BD Difco, Sparks, MD, USA) at 37 °C. All media were supplemented with novobiocin (25 µg/mL; Alfa Aesar, Haverhill, MA, USA) and nalidixic acid (20 µg/mL; MP Biomedicals, LLC, Illkirch, France) to control for growth of extraneous bacteria. Isolates were selected for resistance for differential plating. The SK isolate was selected for resistance to rifampicin (32 µg/mL; Tokyo Chemical Industry Co., Ltd., Portland, OR, USA). The ST and SE isolates were selected for resistance to gentamicin (50 µg/mL; Corning, Manassas, VA, USA). The culture was harvested by centrifugation at 600× *g* for 15 min at 4 °C. The cell pellet was resuspended in sterile, cold phosphate-buffered saline (PBS) and washed twice prior to challenge. The culture’s optical density was measured spectrophotometrically at 625 nm at an absorbance value of 1.30 (SPECTRONIC^®^ 20+ SERIES Spectrophotometers, Thermo Fisher Scientific, Waltham, MA, USA) and estimated at 1.0 × 10^9^ colony-forming units (CFU)/mL relative to an established standard curve. The concentrations of challenge stocks were confirmed by serial dilution on xylose lysine tergitol-4 (XLT-4; Hardy Diagnostics, Santa Maria, CA, USA) agar with added supplement (BD Difco).

### 2.3. Experimental Design and Sample Collection

Chick tray papers were pre-enriched in 10 mL of buffered peptone water (BPW; BD Difco) and incubated overnight at 37 °C. The following day 0.1 mL of pre-enrichment was sub-cultured into 10 mL of Rappaport–Vassiliadis broth (RV; Hardy Diagnostics) at 37 °C overnight. The enrichment was cultured onto XLT-4 without antibiotics and incubated for 18–24 h at 37 °C to verify that chicks were *Salmonella* free. Chicks were randomly divided into six treatment groups of 30 birds each (Table 2 and Table 3).

Chicks were killed by carbon dioxide asphyxiation on D3. Ceca, liver, and spleen samples were aseptically removed. Cecal tonsils and liver samples were snap-frozen in liquid nitrogen and stored at −80 °C until the total RNAs were isolated.

### 2.4. Bacteriological Analysis

There were two sets of XLT-4 plates dependent on the selected antibiotic resistance of each serotype. Samples were sub-cultured onto XLT-4 plates containing novobiocin (25 µg/mL), nalidixic acid (20 µg/mL; XLT-4^NN^) and either rifampicin (32 µg/mL) or gentamicin (50 µg/mL). All samples were incubated for 18–24 h at 37 °C.

Cecal colonization and incidence were measured from 20 chicks per treatment. Livers and spleens were macerated together to measure organ invasion from 20 chicks per treatment. Cecal contents were weighed, and approximately 0.25 g of the contents were serially diluted to 1:10, 1:100, 1:1,000, and 1:10,000 in PBS and spread plated onto XLT-4^NN^ with either rifampicin or gentamicin. All were cultured in BPW overnight at 37 °C before being enriched in RV. The RV cultures were then sub-cultured onto XLT-4^NN^ and incubated 18–24 h at 37 °C. Colonies exhibiting normal *Salmonella* morphology were periodically confirmed by lysine iron agar (LIA; BD Difco), triple sugar iron agar (TSIA; BD Difco) slants, and *Salmonella* O Poly A-I antiserum (BD Difco).

### 2.5. RNA Isolation and Quantitative Real-Time Polymerase Chain Reaction (qRT-PCR)

Total RNA extraction, cDNA synthesis, and quantitative real-time polymerase chain reaction (qRT-PCR) were previously described [47]. Quantification of IL-1β, IL-6, IL-10, and IL-18, and IFN-γ were determined by qRT-PCR using the Applied Biosystems PowerUp™ SYBR™ Green Master Mix (Thermo Fisher Scientific). Primer sequences have been previously reported for all genes except IFN-γ [48,49,50,51,52]. The IFN-γ primers were designed for the current study: (F) 5′ CTTGAGAATCCAGCGCAAAG 3′ (R) 5′ GTTGAGCACAGGAGGTCATA 3′. Each qRT-PCR plate contained target genes and housekeeping gene, β-actin in triplicate and a no-template negative control [53]. The qRT-PCR data were analyzed by the double delta Ct method [54]. The expression of cytokines was calculated as fold change in mRNA levels as compared to the negative control.

### 2.6. Statistical Analysis

Statistical analyses were conducted via a Student’s *t*-test for enumeration, gene expression and chi-square for incidence, using JMP Pro 15 (SAS Institute Inc., Cary, NC, USA). All the data were presented as mean ± standard error of the mean (SEM). A *p*-value of <0.05 was considered significant when compared to the respective positive control. Each trial was replicated twice at different times. Gene expression data were measured from trials 1 and 3.

## 3. Results and Discussion

Colonization resistance is the inability of potentially pathogenic or foreign bacteria to expand due to host microbiota under homeostatic conditions [55]. In trials 1 and 2, competitive exclusion between SK and ST was measured. *Salmonella* was not detected in any of the negative control cultures in trials 1 and 2. As shown in Table 4, ST was not recovered from the cecal contents in birds when SK was administered 1 day before in trial 1. *Salmonella* Kentucky was significantly reduced when ST was administered prior (Table 4). *Salmonella* Typhimurium was significantly reduced when *Salmonella* Kentucky was orally administered 24 h first and in combination with SK in trials 1 and 2 (Table 4 and Table 5). *Salmonella* Kentucky was recovered from every bird challenged (Table 4 and Table 5).

Competitive exclusion of *Salmonella* Enteritidis, another prevalent foodborne strain isolated from humans and poultry, and *Salmonella* Kentucky was also compared in trials 3 and 4. *Salmonella* was not detected in any of the negative control cultures in trials 1 and 2. *Salmonella* Enteritidis was significantly reduced when *Salmonella* Kentucky was administered first and when administered in combination with SK in trials 3 and 4 (Table 6 and Table 7). *Salmonella* Kentucky was significantly reduced when challenged by SE 24 h later and when SE was administered first in Trial 3 (Table 6). *Salmonella* Kentucky and Enteritidis were recovered from all respective cecal enrichments from birds challenged (Table 6 and Table 7).

Research has shown that strains of *Salmonella* can be used to exclude one another through competitive exclusion along with host defense colonization resistance mechanisms. When ST was administered to day-old chicks, it inhibited other *Salmonella* strains from colonizing the alimentary tract [56]. Yang and colleagues measured competitive exclusion in chicks using SE and ST and presented similar results [23]. Similar to these findings, gnotobiotic pigs were protected from pathogenic ST when administered avirulent *Salmonella* Infantis 24 h beforehand [57]. As seen in this study, oral administration of *Salmonella* reduces subsequent colonization of an isogenic isolate in neonatal broilers.

Heterophil populations increase in the chick’s cecal lamina propria from D2 to D4 post-infection with SE and ST [13,58]. An influx of avian host defense peptides includes gallinacins, cathelicidins and liver expressed antimicrobial peptides, which are upregulated during infection, with SE or ST [59,60,61]. *Salmonella enterica* are facultative intracellular bacteria, which means they can persist in macrophages, travel through the bloodstream, and spread systemically [13,62,63]. The incidence of SK in liver/spleen macerations was significantly higher in the SK + ST group compared to other treatments in trial 1 (Table 4). A significantly higher incidence of SK and ST in liver/spleen macerations was measured in the ST → SK group compared to other treatments (Table 5). In trial 3, there was a higher incidence of SK in the livers/spleens compared to the other treatments (Table 6). Newly hatched chicks are more susceptible to systemic translocation of *Salmonella* from the gut barrier due to an underdeveloped immune system, immature microflora, and a relatively sterile gut [64,65,66,67].

**Figure 1:**
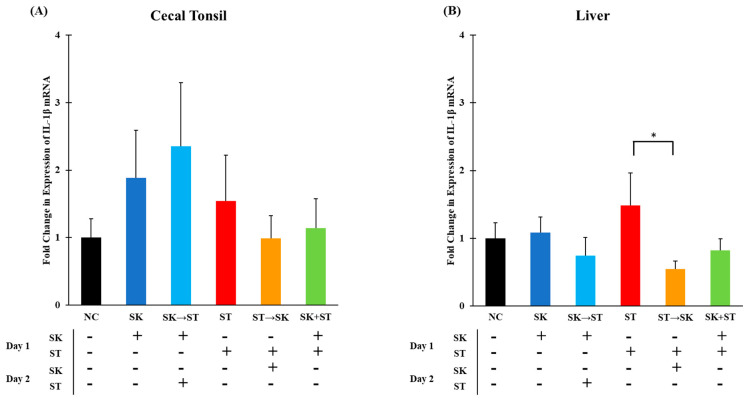
The mRNA levels of interleukin-1β (IL-1β) were not different in cecal tonsils but were significantly higher in the ST group compared to the ST → SK treatment in the liver. Relative mRNA expression of IL-1β gene in (**A**) cecal tonsils and (**B**) liver was determined by quantitative real-time polymerase chain reaction (qRT-PCR) with normalization to the reference β-Actin mRNA levels. *n* = 5 samples per treatment, except for NC where *n* = 4 samples for cecal tonsils and SK → ST *n* = 4 samples for liver. Asterisk (*) on top of the brackets indicates significant differences at *p* < 0.05.

**Figure 2:**
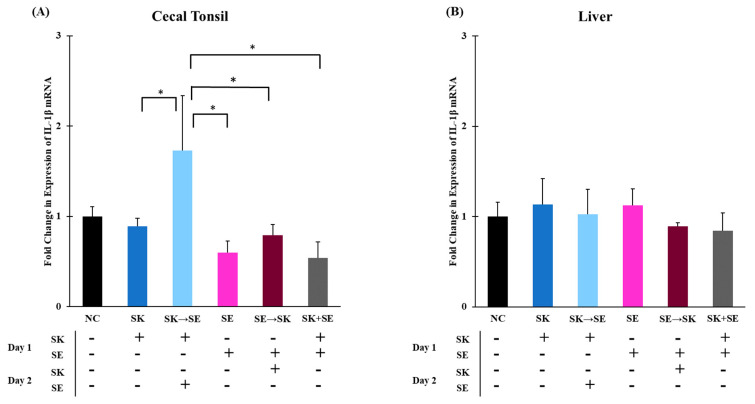
The mRNA levels of IL-1β were higher in the SK → SE treatment as compared to the SK, SE, SE → SK, and SK + SE treatments in the cecal tonsils, but there were no differences in the liver. Relative mRNA expression of the IL-1β gene in (**A**) cecal tonsils and (**B**) liver was determined by qRT-PCR with normalization to the reference β-actin mRNA levels. *n* = 5 samples per treatment, except for SK + SE *n* = 4 samples for cecal tonsils and SK and SK → SE *n* = 4 samples for liver. Asterisk (*) on top of the brackets indicates significant differences at *p* < 0.05.

Interleukin-1β is a key mediator during inflammation and induces the production of chemokines, such as IL-8, to attract specific immune cells [26]. In trial 1, the mRNA levels of IL-1β were not different in the cecal tonsils (Figure 1A). The liver mRNA IL-1β levels were significantly higher in the ST treatment compared to the group given ST followed by SK 24 h later (Figure 1B). In trial 3, the mRNA levels of IL-1β were significantly higher when SK was administered 24 h prior to SE than the treatments of SK, SE, SE followed by SK, and SK and SE combination in the cecal tonsils (Figure 2A). The elevated expression could be due to the consecutive challenge. There were no changes in the liver (Figure 2B). Withanage and colleagues measured a significant increase of IL-1β levels in cecal tonsils of day-old chicks 6–48 h post-infection when challenged with 1.0 × 10^8^ CFU of *Salmonella* Typhimurium [38]. Fasina and colleagues found a significant upregulation in IL-1β mRNA expression in ceca of 5 and 10 days post challenged 4-day-old broilers that were gavaged with 7.8 × 10^6^ CFU/mL of ST [68]. Chranova and colleagues also measured an increase in IL-1β mRNA levels of day-old layer chick ceca when gavaged with 1.0 × 10^6^ CFU of SE [69]. However, changes in IL-1β mRNA levels were not found in the ceca of week-old chicks challenged with 10^8^ CFU of ST in Withanage and colleagues’ earlier studies but was found in the liver 24 h post-infection [11]. Different time point measurements and challenge amounts can affect mRNA expression levels which could be the reason why some of our levels contrast with the literature.

**Figure 3:**
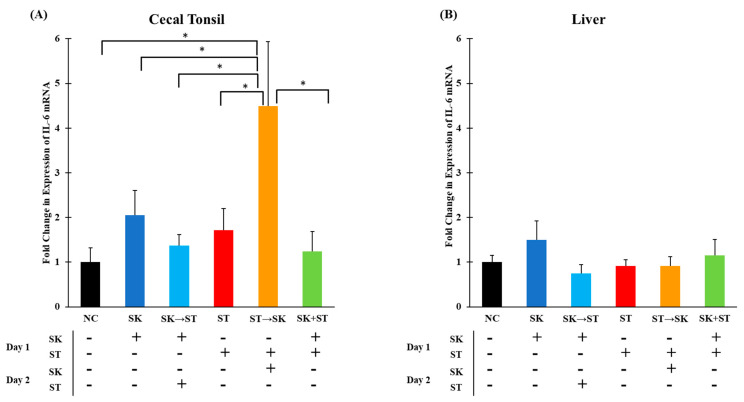
The mRNA levels of IL-6 were significantly higher in the ST → SK treatment in the cecal tonsils but were no differences were found in the liver. Relative mRNA expression of IL-6 gene in (**A**) cecal tonsils and (**B**) liver was determined by qRT-PCR with normalization to the reference β-actin mRNA levels. *n* = 5 samples per treatment, except for NC where *n* = 4 samples for cecal tonsils and NC, SK, and ST → SK were *n* = 4 samples for liver. Asterisk (*) on top of the brackets indicates significant differences at *p* < 0.05.

**Figure 4:**
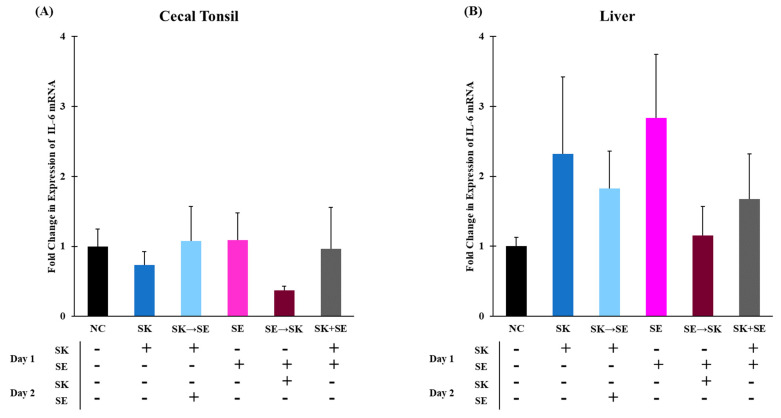
The mRNA levels of IL-6 were not different in the cecal tonsils or liver. Relative mRNA expression of IL-6 gene in (**A**) cecal tonsils and (**B**) liver was determined by qRT-PCR with normalization to the reference β-actin mRNA levels. *n* = 5 samples per treatment, except for SE where *n* = 4 samples for cecal tonsils and SK *n* = 4 samples for liver.

Another initial pro-inflammatory cytokine, IL-6, induces the synthesis of acute phase proteins, such as mannan binding lectin, from hepatic cells to initiate an innate immune response [28,70]. The mRNA levels of IL-6 were significantly higher in the ST followed by the SK group compared to other treatments in cecal tonsils (Figure 3A). An increase in levels could be explained by the consecutive challenge of these two serotypes. There were no significant differences in IL-6 mRNA expression in the liver among the treatment groups (Figure 3B). There were no significant changes in IL-6 expression in the cecal tonsils and liver in trial 3 (Figure 4A,B). Changes in IL-6 mRNA levels were not found in the ceca of day-old chicks challenged with 10^8^ CFU of ST 6–48 h post-infection [38]. Setta and colleagues also measured no changes in IL-6 mRNA levels in the ceca of one-day-old broilers challenged with 10^9^ CFU of SE [12]. In week-old chicks challenged with 10^8^ cfu of ST, IL-6 was not significantly increased until 21 and 28 days post-infection in the ceca and liver [11]. Millet and colleagues measured an acute phase response to ST lipopolysaccharide in whole blood [71]. We did not observe significantly elevated levels of IL-6 expression in the liver; however, expression does not always correlate to protein function.

**Figure 5:**
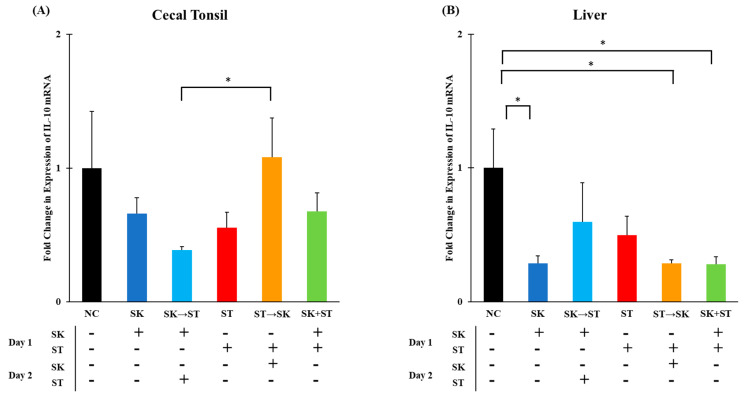
The mRNA levels of IL-10 were significantly higher in the ST → SK treatment than in the SK → ST treatment in the cecal tonsils and significantly higher in the NC treatment than the SK, ST → SK, and SK + ST treatments in the liver. Relative mRNA expression of IL-10 gene in (**A**) cecal tonsils and (**B**) liver was determined by qRT-PCR with normalization to the reference β-actin mRNA levels. *n* = 5 samples per treatment, except for NC where *n* = 4 samples for cecal tonsils and livers. Asterisk (*) on top of the brackets indicates significant differences at *p* < 0.05.

**Figure 6:**
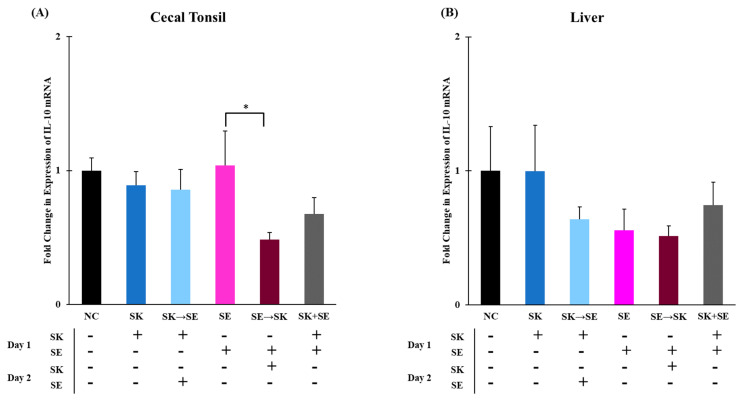
The mRNA levels of IL-10 were significantly higher in the SE treatment than the SE → SK treatment in the cecal tonsils, but there were no changes in the liver. Relative mRNA expression of IL-10 gene in (**A**) cecal tonsils and (**B**) liver was determined by qRT-PCR with normalization to the reference β-actin mRNA levels. *n* = 5 samples per treatment except for SK and SE *n* = 4 samples for liver. Asterisk (*) on top of the brackets indicates significant differences at *p* < 0.05.

Regulatory cytokines, such as IL-10, inhibit the production and secretion of pro-inflammatory cytokines therefore suppressing a T_H_1 response [72,73]. The mRNA levels of IL-10 were significantly higher in the ST followed by SK treatment than in the SK followed by ST treatment in the cecal tonsils (Figure 5A). The levels of IL-10 were higher in the negative control group than the SK, ST followed by SK, and SK and ST combination treatments in the liver (Figure 5B). The mRNA levels of IL-10 were significantly higher in the SE only treatment than the SE followed by SK treatment in the cecal tonsils (Figure 6A). There were no changes in the liver (Figure 6B). The IL-10 levels were downregulated 5 days post ST challenge in the ceca of broiler chicks in Fasina’s previously described experiment [68]. Chranova and colleagues reported mRNA levels for IL-10 were significantly lower in the ceca of the previously mentioned experiment [69].

**Figure 7:**
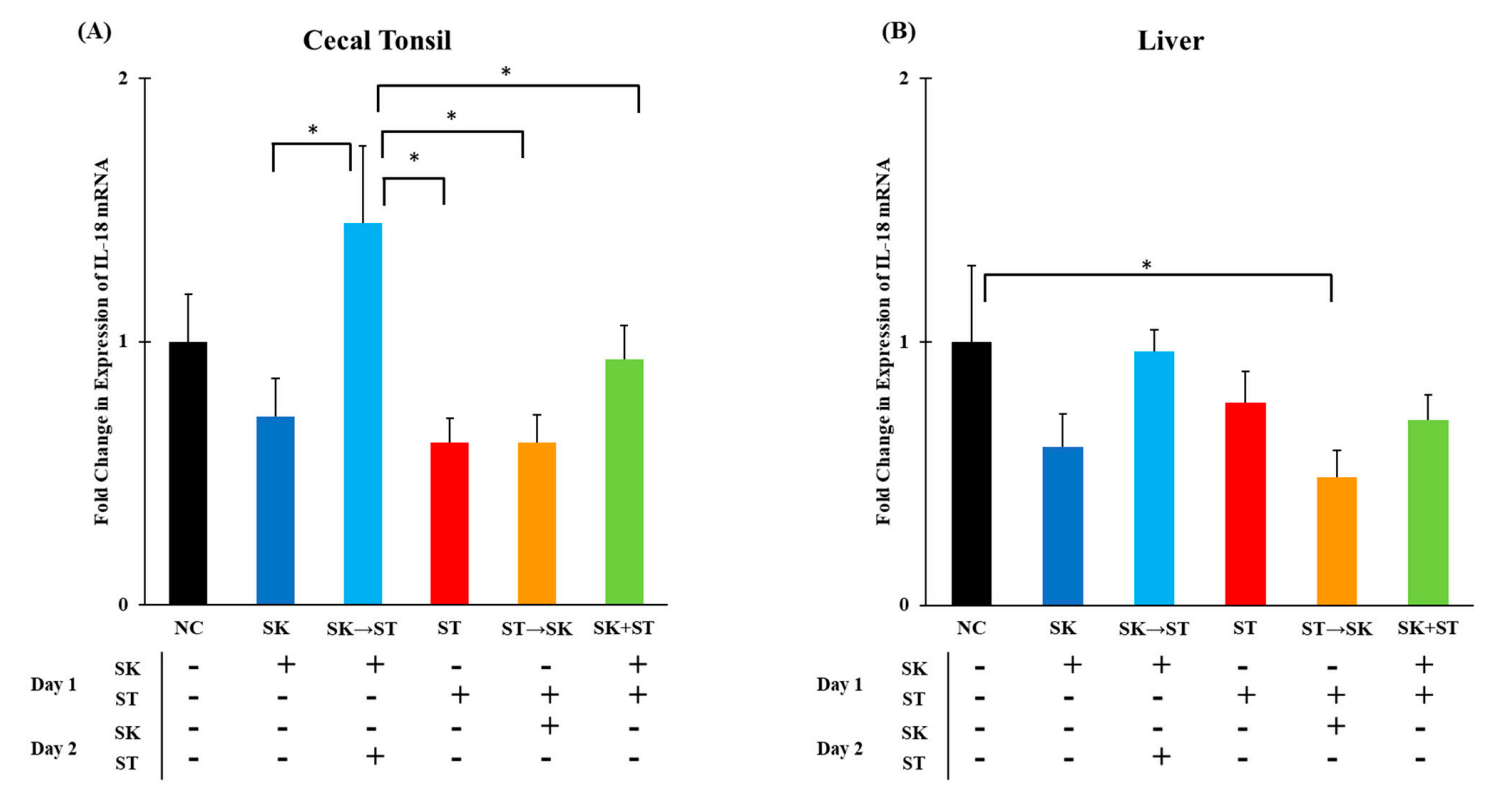
The mRNA levels of IL-18 were significantly higher in the SK → ST treatment than in the SK, ST, ST → SK, and SK + ST treatments in the cecal tonsils. The ST → SK treatment was significantly decreased in the liver as compared to negative control. Relative mRNA expression of the IL-18 gene in the (**A**) cecal tonsils and (**B**) liver was determined by qRT-PCR with normalization to the reference β-actin mRNA levels. *n* = 5 samples per treatment, except for NC where *n* = 4 samples for cecal tonsils and SK → ST *n* = 4 samples for liver. Asterisk (*) on top of the brackets indicates significant differences at *p* < 0.05.

**Figure 8:**
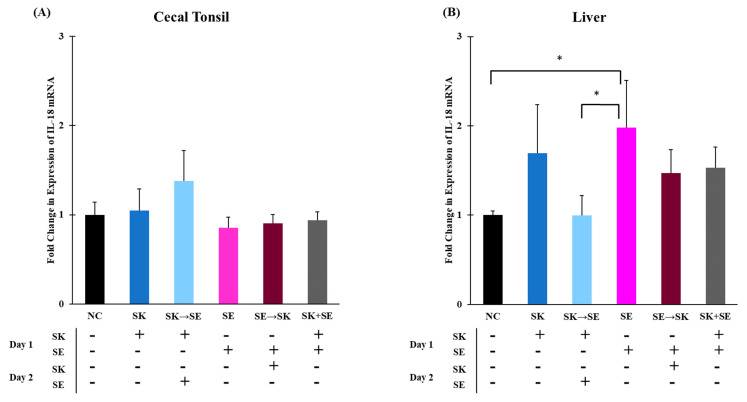
The mRNA levels of IL-18 were significantly higher in the SE treatment than the SK → SE and negative control treatments in the liver, but there were no changes in the cecal tonsils. Relative mRNA expression of the IL-18 gene in (**A**) cecal tonsils and (**B**) liver was determined by qRT-PCR with normalization to the reference β-actin mRNA levels. *n* = 5 samples per treatment, except for SK → SE and SK + SE where *n* = 4 samples for cecal tonsils and SK *n* = 4 samples for liver. Asterisk (*) on top of the brackets indicates significant differences at *p* < 0.05.

Chicken IL-18 is produced by macrophages and induces production of IFN-γ which further mediates T_H_1 cell development [30]. The mRNA levels of IL-18 were significantly higher in the SK followed by ST treatment than in the SK, ST, ST followed by SK, and SK and ST combination treatments in the cecal tonsils (Figure 7A). Levels of IL-18 were significantly higher in the negative control group compared to the ST followed by SK treatment in the liver (Figure 7B). The mRNA levels of IL-18 were significantly higher in the SK followed by SE treatment than the SK only, SE only, SE followed by SK, and SK and SE combination treatments in the cecal tonsils (Figure 8A). There were no changes in the liver (Figure 8B). Berndt and colleagues measured a significant increase in IL-18 mRNA levels in the ceca of chicks gavaged with 1.0 × 10^7^ CFU of SE or ST at a peak 2 and 4 days post-infection [74]. Secretion of IL-18 is important for a later adaptive immune response to produce IFN-γ [30].

**Figure 9:**
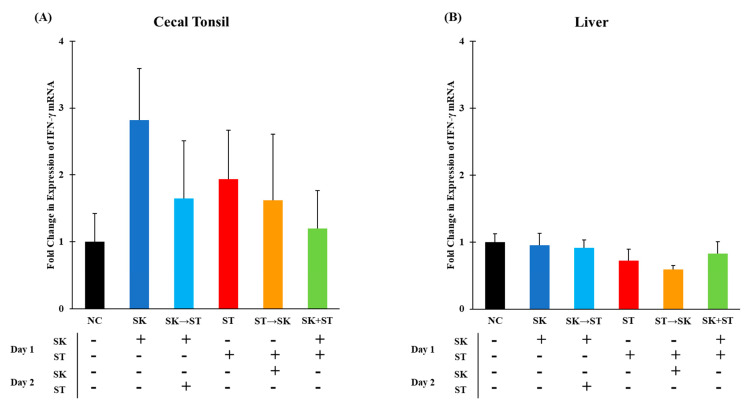
The mRNA levels of gamma interferon (IFN-γ) were not different in the cecal tonsils or liver. Relative mRNA expression of IFN-γ gene in (**A**) cecal tonsils and (**B**) liver was determined by qRT-PCR with normalization to the reference β-actin mRNA levels. *n* = 5 samples per treatment, except for NC, SK, SK → ST, ST and SK + ST where *n* = 4 samples for cecal tonsils and ST → SK *n* = 4 samples for liver.

**Figure 10:**
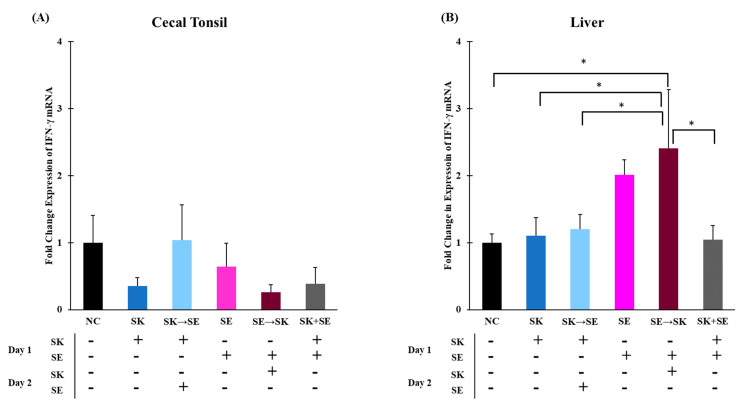
The mRNA levels of IFN-γ were not different in the cecal tonsils, but were significantly higher in the SE → SK group than the NC, SK, SK → ST and SK+SE liver treatments. Relative mRNA expression of IFN-γ gene in (**A**) cecal tonsils and (**B**) liver was determined by qRT-PCR with normalization to the reference β-actin mRNA levels. *n* = 5 samples per treatment, except for NC, SK, SK → ST, ST and SK + ST where *n* = 4 samples for cecal tonsils and ST → SK *n* = 4 samples for liver. Asterisk (*) on top of the brackets indicates significant differences at *p* < 0.05.

Chicken IFN-γ is primarily produced by T_H_1 lymphocytes and natural killer cells and is driven by the production of IL-12 and IL-18 for a later immune response [74,75]. Expression of IFN-γ is critical to the host immune response to intracellular pathogens because it activates macrophages, which increases their ability to kill [76]. There were no significant changes in IFN-γ expression in the cecal tonsils and liver in trial 1 (Figure 9A,B). The mRNA levels of IFN-γ were not different in the cecal tonsils (Figure 10A). Levels of IFN-γ were significantly higher in the SE → SK group compared to the NC, SK, SK → SE, SE, and SK+SE groups in the liver (Figure 10B). Gamma interferon expression is increased by signals from pro-inflammatory or T_H_1 cytokines such as IL-6 and IL-18 in an adaptive immune response [74,77]. Berndt and colleagues measured a significant increase IFN-γ mRNA levels in the ceca up to 4 days post-infection [76].

Cytokine mRNA expression measured by (qRT)-PCR does not always correlate with protein levels, but it is a sensitive method [78]. An increase in cytokine expression has been found as early as 12 h post-infection [35]. Here we measured cytokine mRNA expression 24–48 h post-infection and the responses could have had an earlier or later expression pattern. Broiler chick ceca are colonized by *Salmonella* quicker than the spleen and liver which could explain the variable results of our mRNA levels [55]. The cecal tonsils are a part of the gut-associated lymphoid tissues and are a local site for immune responses against enteric bacteria [79]. The liver produces acute phase proteins in response to pro-inflammatory cytokines [80]. There was a lower incidence of *Salmonella* in the liver/spleen macerations, therefore, it may be that not all the livers were affected like the ceca. However, soluble factors from the gastrointestinal tract can affect the liver, so that could explain the results seen [37]. Cheeseman and colleagues measured an increase of IFN-γ in the spleen compared to the ceca in birds challenged with SE, indicating differences in immune gene expression across organs [81]. Withanage and colleagues found initial cytokine detection was greater in the liver before the spleen indicating a more rapid response [38]. The differences in cytokine gene expression suggest differences in *Salmonella* subspecies’ interactions and pathogenesis [82].

The current research focused on the characterization of an immune response in the cecal tonsils and liver during a concurrent infection. Intestinal cytokine responses are stimulated by foreign or pathogenic bacteria [83]. *Salmonella* may not be pathogenic to the avian host, but it can persist and colonize the cecal lumen of chickens, which would allow shedding into the environment [84]. As previously mentioned, *Salmonella* Kentucky was isolated from fewer human cases, than ST and SE [8]. Yet, SK was isolated from more chicken carcasses than ST and SE combined [3]. In conclusion, the data presented show that the oral administration of *Salmonella* Kentucky reduced subsequent colonization of Enteritidis and Typhimurium in neonatal broilers. The current study shows expression of cytokines were affected by consecutive challenges indicating immune function could be altered during competitive exclusion. A subunit vaccine exploiting SK’s mechanisms to colonize and persist in chickens could benefit public health and agricultural sectors.

## Figures and Tables

**Table 1:** Source of *Salmonella* serotypes.

Bacteria	Strain	Source of Strain	Reference
S. Enteritidis	Primary poultry isolate, #97-11771	National Veterinary Services Laboratory Ames, Iowa, USA	[44]
S. Kentucky	Broiler field isolate	Southern USA Farm	[45]
S. Typhimurium	Primary poultry isolate	National Veterinary Services Laboratory Ames, Iowa, USA	[46]

**Table 2:** Experimental design for trials 1 and 2 (*n* = 30 chicks/treatment).

Treatment	NC	SK	SK → ST	ST	ST → SK	SK + ST
D0	Place chicks					
D1 (Challenge)	PBS	10^4^ CFU SK	10^4^ CFU SK	10^4^ CFU ST	10^4^ CFU ST	10^4^ CFU SK + ST
D2 (Challenge)	PBS	PBS	10^5^ CFU ST	PBS	10^5^ CFU SK	PBS
D3 (Kill)	Collect ceca, liver, and spleen tissues.					

All chicks received 0.5 mL of PBS or challenge. NC, negative control; SK, *Salmonella* Kentucky; ST, *Salmonella* Typhimurium.

**Table 3:** Experimental design for trials 3 and 4 (*n* = 30 chicks/treatment).

Treatment	NC	SK	SK → SE	SE	SE → SK	SK + SE
D0	Place chicks					
D1 (Challenge)	PBS	10^4^ CFU SK	10^4^ CFU SK	10^4^ CFU SE	10^4^ CFU SE	10^4^ CFU SK + SE
D2 (Challenge)	PBS	PBS	10^5^ CFU SE	PBS	10^5^ CFU SK	PBS
D3 (Kill)	Collect ceca, liver, and spleen tissues.					

All chicks received 0.5 mL of phosphate-buffered saline (PBS) or challenge. NC, negative control; SK, *Salmonella* Kentucky; SE, *Salmonella* Enteritidis.

**Table 4:** Trial 1 colonization and incidence of cecal contents and organ invasion in macerated liver and spleen (L/S).

Treatment ^2^	SK Ceca Log_10_(cfu/g)	SK Ceca Incidence	SK L/S Incidence	ST Ceca Log_10_(cfu/g)	ST Ceca Incidence	ST L/S Incidence
NC	0.00	0/20	0/20	0.00	0/20	0/20
SK PC	5.36	20/20	2/20	0.00	0/20	0/20
SK → ST	**4.69 ^1^**	20/20	1/20	**0.00 ^1^**	0/20	0/20
ST PC	0.00	0/20	0/20	4.90	20/20	3/20
ST → SK	**4.40 ^1^**	20/20	1/20	5.35	20/20	0/20
SK + ST	5.58	20/20	**5/20 ^1^**	**3.74 ^1^**	20/20	2/20

^1^*p* < 0.05 (*p*-values were calculated in comparison to the respective positive control). ^2^ *n* = 20 samples/treatment. NC, negative control; PC, positive control; SK, *Salmonella* Kentucky; ST, *Salmonella* Typhimurium; L/S, liver and spleen macerated.

**Table 5:** Trial 2 colonization and incidence of cecal contents and organ invasion in macerated liver and spleen (L/S).

Treatment ^2^	SK Ceca Log_10_(cfu/g)	SK Ceca Incidence	SK L/S Incidence	ST Ceca Log_10_(cfu/g)	ST Ceca Incidence	ST L/S Incidence
NC	0.00	0/20	0/20	0.00	0/20	0/20
SK PC	5.24	20/20	1/20	0.00	0/20	0/20
SK → ST	5.20	20/20	3/20	**2.23 ^1^**	20/20	0/20
ST PC	0.00	0/20	0/20	4.79	20/20	1/20
ST → SK	**4.60 ^1^**	20/20	**9/20 ^1^**	5.02	20/20	**9/20 ^1^**
SK + ST	**4.79 ^1^**	20/20	4/20	**3.75 ^1^**	20/20	2/20

^1^*p* < 0.05 (*p*-values were calculated in comparison to the respective positive control). ^2^ *n* = 20 samples/treatment. NC, negative control; PC, positive control; SK, *Salmonella* Kentucky; ST, *Salmonella* Typhimurium; L/S, liver and spleen macerated.

**Table 6:** Trial 3 colonization and incidence of cecal contents and organ invasion in macerated liver and spleen (L/S).

Treatment ^2^	SK Ceca Log_10_(cfu/g)	SK Ceca Incidence	SK L/S Incidence	SE Ceca Log_10_(cfu/g)	SE Ceca Incidence	SE L/S Incidence
NC	0.00	0/20	0/20	0.00	0/20	0/20
SK PC	5.94	20/20	**7/20 ^1^**	0.00	0/20	0/20
SK → SE	**5.07 ^1^**	20/20	2/20	**2.89 ^1^**	20/20	0/20
SE PC	0.00	0/20	0/20	4.52	20/20	0/20
SE → SK	**3.90 ^1^**	20/20	0/20	4.21	20/20	0/20
SK + SE	5.40	20/20	2/20	**3.92 ^1^**	20/20	2/20

^1^*p* < 0.05 (*p*-values were calculated in comparison to the respective positive control). ^2^ *n* = 20 samples/treatment. NC, negative control; PC, positive control; SK, *Salmonella* Kentucky; SE, *Salmonella* Enteritidis; L/S, liver and spleen macerated.

**Table 7:** Trial 4 colonization and incidence of cecal contents and organ invasion in macerated liver and spleen (L/S).

Treatment ^2^	SK Ceca Log_10_(cfu/g)	SK Ceca Incidence	SK L/S Incidence	SE Ceca Log_10_(cfu/g)	SE Ceca Incidence	SE L/S Incidence
NC	0.00	0/20	0/20	0.00	0/20	0/20
SK PC	6.74	20/20	3/20	0.00	0/20	0/20
SK → SE	6.62	20/20	1/20	**2.48 ^1^**	20/20	0/20
SE PC	0.00	0/20	0/20	5.10	20/20	0/20
SE → SK	**3.97 ^1^**	20/20	2/20	5.20	20/20	0/20
SK + SE	6.33	20/20	1/20	**3.98 ^1^**	20/20	0/20

^1^*p* < 0.05 (*p*-values were calculated in comparison to the respective positive control). ^2^ *n* = 20 samples/treatment. NC, negative control; PC, positive control; SK, *Salmonella* Kentucky; SE, *Salmonella* Enteritidis; L/S, liver and spleen macerated.

## Data Availability

Not applicable.
